# Long-term mentoring relationships in undergraduate longitudinal general practice tracks – a qualitative study on the perspective of students and general practitioners

**DOI:** 10.1080/10872981.2022.2149252

**Published:** 2022-12-04

**Authors:** Anna Scholz, Vera Gehres, Anne Schrimpf, Markus Bleckwenn, Tobias Deutsch, Anne-Kathrin Geier

**Affiliations:** Department of General Practice, Faculty of Medicine, Leipzig University, Leipzig, Germany

**Keywords:** General practice, undergraduate medical education, mentoring medical students, general practice track, qualitative study

## Abstract

**Background:**

Longitudinal general practice tracks have been established in medical faculties in Europe and worldwide to attract more graduates to general practice careers. In many programs, long-term mentoring relationships play an important role in providing students with positive role models, regular practical experiences, and acquisition of clinical skills in a community context. However, little is known about students’ and general practitioner mentors’ expectations, experiences, challenges, and ideas for improvement within these long-term mentoring relationships in general practice in our medical education system.

**Methods:**

Qualitative study based on semi-structured interviews with 15 students and 13 mentors. Interviews were audio-recorded and transcribed verbatim. MAXQDA was used for data analysis, following a mixed deductive/inductive approach.

**Results:**

Both groups had few and rather unstated expectations, particularly regarding their relationships. Consequently, expectations were often not clearly communicated. Nevertheless, a high level of satisfaction and good opportunities for teaching were achieved for both sides. The evolving familiarity facilitated a positive learning environment. Students valued independent medical tasks continuously adjusted to their current abilities. However, some felt a reluctance to demand their mentor’s time and consideration. Conversely, the mentors criticized a lack of initiative from some of the students and wished that they would get more actively involved. Students, in contrast, wished for more guidance at the start of the project and joint events to deepen the relationship.

**Conclusions:**

With this study, we gained detailed insights into and understanding of the nature of long-term relationships between students and mentors. Points for improvement revealed included: 1) education of both participating groups on the goals and benefits of mentoring, including binding expectations for the participants; 2) intensified support and training of teaching physicians; 3) structured and accompanied establishment of initial contact between mentor and mentee; and 4) encouraged additional shared (teaching) time, individualized timing, and intensification, if desired.

## Introduction

Many medical schools, nationally and worldwide, have implemented curricular and extracurricular general practice projects to combat physician shortages in this field [1–4]. The decision of pursuing a career in general practice depends, among others, on perceived and actual characteristics of the specialty, sociodemographic factors (e.g., female sex, older age), and experiences during medical education [3,5–11]. Regarding possible contributions from medical schools and faculties, there is broad evidence that good quality teaching, positive role models, and early and continuous exposure to general practice content as experienced in longitudinal programs have the potential to increase the number of graduates choosing general practice careers [3–5,7,12–14].

Good quality teaching in the ambulatory care setting depends on several factors, such as student’s and general practitioner (GP) mentor’s motivation, enthusiasm, and enjoyment [15,16]. Students benefit from a teaching style that is flexible and adapted to their level of knowledge [15–17], as well as from the opportunity for diverse patient contacts [9], practical exercise [4,9], and autonomy in patient encounters [18,19]. A respectful and trusting relationship between student and preceptor is among the fundamental prerequisites for successful learning [18,20]. A particularly close relationship between student and teacher can be achieved by one-to-one mentoring. Mentoring is characterized by a transfer of knowledge, exchange of experience, and a long-term relationship, so additional benefits arise for the student in terms of individual advice, support, and guidance [21–23]. The role of mentoring in medicine in general and in general practice specifically has received much attention in current research [24–26].

At the Department of General Practice of the Leipzig University, the ‘Leipziger Kompetenzpfad Allgemeinmedizin’ (‘Leipzig Competency Pathway for General Practice’, LeiKA) has been integrated into medical education as an optional longitudinal curriculum since 2016 [27]. It offers 30 slots per year for interested first year medical students. Enrolled students participate in this program through their entire undergraduate medical education, which lasts 6 years (2 years of basic science, 3 years of clinical science, 1 year clinical rotations) [28]. One pillar of the extracurricular LeiKA teaching project is the individual mentorship between students and their GP mentors. The student attends their GP mentor’s community practice four days per year and experiences their daily routine. This enables the students to have in-depth insights and a realistic and enhanced understanding of ambulatory care, meanwhile being supported and accompanied by their personal GP mentors.

GP mentors are university-affiliated teaching physicians. They are contractually bound to the university and carry out the curricular compulsory courses in the outpatient area. They receive didactic training at the beginning of their activity and are remunerated for their work. (Voluntary) trainings and meetings are held regularly. The supervision of students in the longitudinal LeiKA project is additional and voluntary, for which they also receive financial compensation. The original German name for the LeiKA-mentorship is ‘LeiKA-Patenschaft’, which translates into LeiKA-godparenthood. Compared to other preceptorships, this might implicate a more personal component in supervision and companionship. The concept encompasses not only clinical mentoring, but leaves room for individual interpretation by the participants.

Many experiences with mentoring and preceptoring in longitudinal programs in undergraduate medical education have been made in Anglo-Saxon countries, e.g., in the context of longitudinal integrated clerkships [20,29–31]. However, there are substantial differences in medical education worldwide. Insights from this specific teaching format cannot automatically be transferred to other longitudinal programs and projects that differ in extent and design. Further, the important role of mentors and role models in primary care career choice has again been highlighted in a recent review, although the authors highlighted the fact that consistent definitions are currently lacking [32].

After five years of running the project, the time had come to evaluate whether our idea of evolving partnerships, professional insights, support, and the establishment of positive role models had become reality, what the project administration could offer to support the participants, and which implications could be drawn for current and future programs. Therefore, the aim of this research project was to investigate individual mentee-mentor relationships from the participants’ point of view. Qualitative interviews were used to answer the following research questions: 1) What were the initial expectations of students and GP mentors taking part in the project, and have those expectations been met? 2) How is the mentorship configured in everyday practice life? 3) How did the relationships evolve over time? 4) What are needs for support and wishes for improvements? 5) What implications for adjusting longitudinal programs can be derived?

## Materials and methods

### Sampling

All GP mentors (n = 55) and all students (n = 77) from the 2016 to 2018 cohorts were considered key informants (inclusion criteria) and invited to take part in the study. These students and GP mentors had, for the most part, been enrolled in the project for 3–5 years at the time of the study and could draw on a certain level of experience and shared visit days. Students and GP mentors from the cohorts 2019 and younger were excluded.

Students were contacted via email in February 2021. Interviews were started on a first-come first-served basis. In the course of the interviews, the participants were selected according to sociodemographic characteristics (sex, age, foreign background, completed vocational training, geographic location of GP mentors’ practice, number of semesters in LeiKA, and cross entry in later study years) to balance and obtain a heterogeneous spectrum. As the first recruitment wave mainly recruited German female students, in a second recruitment wave, students from underrepresented groups (e.g., male students, students with foreign background) were especially encouraged to participate.

GP mentors were contacted via mail in June 2021. Considered characteristics to maximize heterogeneity were sex, age, geographic location of GP mentors’ practice, professional, teaching experience, and number of students supervised in LeiKA.

Interviews were stopped as soon as saturation appeared in the analysis.

### Ethics committee approval

The study was carried out in accordance with the Declaration of Helsinki and the study protocol was approved by the research ethics committee of the Leipzig University (reference number 148/21-ek). All participants received written information about the research project and privacy policy before signing an informed consent form containing detailed description of aims and procedures of the study. They did not receive an incentive for their participation.

In order to protect the participants’ confidentiality interviews were conducted by a researcher not involved in administrating the project or in teaching, supervising, or evaluating students. The data were analyzed pseudonymously to make it difficult to identify the students and GP mentors.

### Procedure

Individual, semi-structured qualitative interviews were conducted from April to December 2021. There was no relationship between the interviewer and the potential interviewee. The interviews were planned to last about 30 minutes and were conducted face-to-face in the GP mentor’s practice or via videoconference using BigBlueButton. The interview guideline was self-developed in the Department of General Practice of the Leipzig University by an interdisciplinary research team (medical scientists, psychologist, medical student, and GP trainees) based on an extensive literature search aimed at identifying relevant factors for relationships in mentoring [16,19,33–36]. It underwent a multi-stage revision process and pretesting with a GP mentor and medical student. A summarized guideline version is shown in Table 1. A complete version is presented in Appendix 1. All interviews were audio recorded and pseudonymously transcribed verbatim. Minor adjustments to the guideline were made according to previous interviews and simultaneous data analysis.

**Table 1.** Summarized interview guideline.

What **expectations** did you initially have of the mentorship?
To what extent were these expectations initially **communicated**?
How would you currently describe the **relationship**?
How would you **define mentorship** as part of the LeiKA project?
To what extent have your initial expectations of the mentorship been met?
What do you **wish** for from your GP mentor/your student as part of the LeiKA project?
How could the relationship between you and your GP mentor/your student be **supported**?
Where do you see the overall **benefit** from a long-term mentorship between student and GP?

### Data analysis

The software MAXQDA 2020 (Verbi GmbH, Berlin, Germany) was used for coding and qualitative content analysis according to Kuckartz [37], following a mixed deductive and inductive approach: main categories were deductively identified in advance in accordance with the main topics of the interview guideline. These were expectations, communication of expectations, mentorship’s realization, definition of a mentorship, wishes, improvements, and benefits. Inductive categories were derived during the line-by-line coding, either indicating new main themes or expanding the initial main themes with second-order categories. Data were analyzed in parallel with conducting the interviews, resulting in small changes in the focus of the questions during the course of the interviews. All interviews were iteratively revised with the inductively generated categories after the initial coding was completed.

Data collection was stopped as soon as saturation appeared, indicated by data redundancy and the emergence of no new themes in the last two interviews of each group (students and GP mentors) [38–40].

Reliability of the coding was ensured through an inter-coder agreement: a second researcher (GP trainee working as medical scientist) repeated the coding independently using the descripted coding tree. An inter-coder agreement of 94.1% for students and 94.4% for GP mentors was achieved. Discrepancies and results of the coding were additionally discussed with the whole interdisciplinary research group (medical scientists, psychologist, medical student, and GP trainees). The coding tree with main and sub-categories, definitions for categories, and sample quotes are available on request.

## Results

In total, 15 student interviews and 13 GP mentor interviews were conducted. Student interviews lasted 20 to 39 minutes (30 min ± 5 min), and those with GP mentors lasted 18 to 40 minutes (24 min ± 6 min). Sample characteristics are displayed in Table 2.

**Table 2.** Sample characteristics.

		students (n = 15)	GP mentors (n = 13)
Female sex		n = 11 (73.3%)	n = 9 (69.2%)
Age in years (mean ± SD, Min/Max)		23.0 ± 1.4, 22/27	55.4 ± 10.3, 36/70
Number of years in medical school:	3 years	n = 4	N/A
	4 years	n = 8	N/A
	5 years	n = 3	N/A
Number of semesters in LeiKA (mean ± SD, Min/Max)		7.4 ± 1.6, 4/10	7.5 ± 1.8, 5/10
Practice located in rural area		n = 9 (60%)	n = 9 (69.2%)

### Definition of a mentorship

#### Students’ definition of a mentorship

When students were asked about their principal idea of mentorship, most had difficulty defining their concept of it. Many students mentioned the mentor’s function as a supervisor who provides support and guidance with respect to medical studies and career plans. However, some students extended their idea of mentorship to private and personal exchanges. The long-term nature of the mentorship was regarded as the basis for better mutual understanding and trust. In addition, the students understood mentorship as mutual, free of obligations, but with an emphasis on help and support in times of need.

*“Well, that is a good question, because I don’t really know how I would define a mentorship. Maybe, a mentorship has definitely very long-term aspects, so there is a degree of long-term perspective […], well, in any case it is set for several years. […] And otherwise, well, that you somehow get to know each other […]. And somehow [to have] this contact person, that might be […] a bit like a mentor. Although the term might also convey […] more professional [exchange], help, support.” (Student No. 8, female)*

#### GP mentors’ definition of a mentorship and their role as a mentor

Analogous to the students, the mentors were asked about their definition of a mentorship. The GP mentors valued the mentorship as an opportunity to provide support during a phase of the students’ lives, participating in the development and progress of the students. GP mentors named the long-term relationship as a distinctive feature in contrast to other teaching formats. They perceived themselves as contact persons and role models and some emphasized the commitment, they in turn were making. According to the GP mentors, a mentorship was based on a conscious decision made by both sides, and each one had to openly engage with their counterpart.

*“So, a mentorship means […] that I really accompany a phase of [the student’s] life. And that in good days and in bad days. I would see it the same way in the course of study. So, I say if one […] fails an exam or if something like that is [happening]. So, you can’t reduce that [to the visit days]. Then it’s not fun. So, it has to be a togetherness […]. [The students] should accompany the practice and we should accompany the course of study. […] and then they should always know, I have something in my hand here, and that is something I can refer to, so, if I have problems, I can also go and ask. […] But it’s not an obligation. So, I wouldn’t see that as an obligation that students have to show up here every month and say, I’m doing this and that. That’s not my job either, yeah, but I have to have the door open.” (Physician No. 3, female)*

When asked explicitly about their own understanding of their role, GP mentors identified themselves as role models, wishing to impart medical values and attitudes as well as formal and informal medical knowledge. Further, they saw themselves as contact persons available in case of problems, concerns, or questions to support and advise the students during their studies, independent of occurring problems or difficulties.

*“[I see my task as a GP mentor as] maybe being there for him outside of a typical teacher. So, also little tips and tricks, like it is in real life, something like that. Apart from that, all the tasks that you want to pass on to the young man or woman if you want to pass on your profession.” (Physician No. 2, female)*

### Expectations

#### Students’ expectations

The students were asked about their initial expectations of the mentorship. A substantial proportion had not thought about the mentoring relationship before the interview and thus had difficulty formulating expectations. For many, the prospect of patient contact and practice visits had been the primary motivation to apply for the project, rather than the provision of a long-term clinical preceptor. Students who formulated explicit expectations towards the relationship had been looking for a role model and contact person who would accompany them during their studies. For them, the long-term nature of the relationship was as important as the expectation of high-quality, tailored teaching.

*“[My expectations were] that I could learn a lot, that I can simply gain practical experience. But also, that there would be a good relationship between me and my GP mentor and that I could turn to him for help with other questions, if there was something in my studies or with my MD thesis or whatever. Because it is simply a contact person who, well, somehow knows his way around in everyday medical life and whom I can ask things.” (Student No. 2, female)*

#### GP mentors’ expectations

When asked about their expectations placed on their assigned students, most GP mentors had few or very vague notions, especially regarding interaction and togetherness. The general impression was conveyed that most GP mentors were reflecting on their expectations for the first time at the time of the interview. Several expected students to show an explicit interest in general practice, a willingness to be involved in everyday procedures and to connect with the team, regular and reliable participation in the project, and the readiness to learn.

*“To be honest, I had few expectations of the students. Of course, I hoped that they would sign up for this project because they wanted to and not because there were still places available […], but because there was at least partial interest in general medicine. And they also wanted to back this up with practical experience.” (Physician No. 1, female)*

### Communication of expectations

#### Communication of expectations from students’ perspective

Further, we examined to what extent initial expectations had been communicated between students and mentors. Half of the students had the opportunity to talk about mutual expectations with their mentors, albeit to varying degrees. There was little discussion of the format and frequency of communication outside of regular visits. While some had extensive conversations about teaching content, structure of the practice visits, and the transfer of knowledge, others reported limited exchanges or none due to the following reasons: 1) students felt unprepared when being asked by their GP mentor about their expectations and had not previously thought about them, 2) the perceived GP mentor’s authority discouraged students from forming expectations and being proactive, or 3) the time provided by the GP mentor did not allow for extensive talks about expectations.

*“I never directly said that I would like to do this and that. She [my GP mentor] just said that if I ever needed anything or would like to do something, I should just let her know beforehand or in between, and then we could talk it through or do it.” (Student No. 1, female)*

#### Communication of expectations from GP mentors’ perspective

When we examined the extent to which expectations had been communicated, some GPs reported initial talks while others had gone straight to day-to-day business. Where conversations had taken place, they included mutual acquaintance, exchange of mutual motivations to participate, planned teaching content, and structure of the practice visits. In contrast, there had been little discussion about future extension and means of communication. Some GP mentors valued the welcome reception organized by the LeiKA administration as a good opportunity to get in touch outside of everyday practice and said it provided communicative support. However, this meeting was not compulsory and not all students and GP mentors participated. In addition, there were GP mentors who did not communicate any expectations to the student and one GP mentor said that he would have expected the student to initiate this.

*“Well, I think that we communicated in general why we were doing it, what our expectations were. And in the end, we told the students that we were happy that they were interested and that they were very welcome. Yes, the welcome was always expressed, yes. And actually, the willingness to participate in the student development as well.” (Physician No. 5, female)*

### Fulfillment of expectations and satisfaction with the mentorship

#### Students’ fulfillment of expectations and satisfaction with the mentorship

Students were then asked to what extent their expectations were met and how satisfied they were with the mentorship in general. Most students were highly satisfied with the relationship, the insights gained, and the skills learned, though not always right from the start. Apart from the time it took for the relationship to develop, there were also substantial limitations due to the interruption by the COVID-19 pandemic. However, many students named limitations in terms of connectedness and teaching style. Some students mentioned a limited transfer of independent tasks within the practice (e.g., blood sampling, joint anamnesis) and only a few referred to their mentors quizzing them about their knowledge. A few students wished the personal exchange to be more intense and regular. Nevertheless, students emphasized the special character of mentoring compared to a regular preceptorship, except for one student who perceived the mentorship as an internship spread over five years.

*“The only thing that, I think, has not been fulfilled is a little bit of this private thing. […] Apart from that, it has actually been completely fulfilled. […] It’s really cool how they take care of me and that this is also part of the mentorship. But on the other level, this personal exchange, that just isn’t there.” (Student No. 14, female)*

#### GP mentors’ fulfillment of expectations and satisfaction with the mentorship

Most GP mentors were satisfied with the project. Highly motivated and interested students were assigned to them, the integration of the student into everyday practice worked well, and the relationship developed according to their expectations. It was noted that the best possible result was achieved considering the given circumstances (limited time capacity, few practice visits, and varying interpersonal relationships). Those who saw limitations named especially the lack of regular contact and a too loose connection to each other. While some criticized a lack of commitment and investment on the part of the students, others blamed the limited number of practice visits. One GP mentor expressed negative attitudes towards the students’ medical skills and perceived interest. A few GP mentors were self-critical and wondered if they should have taken more initiative to improve their relationship. Several GP mentors were optimistic and perceived the relationship to be still in an emerging developmental process.

*“So, I think the expectations [have been] already fulfilled in a sense that they [the students] then also were really willing [to learn]. […] Well, as I said, the expectations of long-term guidance, I would have expected a bit more, that more would come back from the student, […] that is missing a bit. Apart from that, the way a visit day is proceeding now, here the expectations have actually already been fulfilled, in that one can already impart a lot and that there are many questions [by the student]. […] Yes, as I said, […] [the relationship] could actually be even closer.” (Physician No. 4, male)*

### Practical realization of mentorships

#### Structure and procedure of clinical visits

##### Structure and procedure of clinical visits from students’ perspectives

When asked how clinical visits were structured and arranged in everyday practice, students described a high variety in knowledge transfer, ranging from mere observation to performing independent tasks. In most cases, a high level of independence in treating patients under supervision was achieved, either from the start, or students grew into it over time. For others, more independence in their activities had been scheduled but had not yet been achieved, due to the interruption by the COVID-19 pandemic or GP mentors’ uncertainties regarding the delegation of tasks. Importantly, students fundamentally appreciated independence. Although some students felt overloaded at the beginning when being faced with their own patients, the transfer of responsibility was highly valued over time. Students who spent extra time at their GP mentor’s practice reported an increased gain of independence, more experiences, and a closer relationship to their GP mentor.

*“But I also thought that [doing things on my own] was really cool, because I tended to only watch at the beginning, but then at some point I also participated […] and then [my GP mentor said] directly: […] You do it next time and I will take a look. Which I also think is really cool, because I think that is how you learn the most. Well, and during the medical clerkship it was really like this: Well, now just do it on your own and we will be at the front [desk], if you have anything, come and ask. And I think that is really cool, because that way you notice a little more where your deficits are and where you still have to learn somehow and also how to really deal with patients” (Student No. 14, female)*

Students mentioned a few points as not being beneficial for their learning. Two students described their seating position in the room as unfavorable and two were unable to treat patients independently due to a lack of a separate space. One student had the impression that treating patients independently was not feasible because it took up too much time. Other students were dissatisfied with the GP mentor’s recognition and appreciation of their knowledge as well as its increase over the course of the mentorship. Further, a few students reported a lack of additional opportunities to have conversations with their GP mentor beyond the regular daily patient consultations. Since most students had not actively expressed desires for what they explicitly wanted to learn during the mentorship, the quality of teaching depended mostly on the GP mentor’s initiative to challenge the students.

##### Structure and procedure of clinical visits from GP mentors’ perspectives

When the GP mentors were asked how the clinical visits were structured, the teaching situation showed great variations, ranging from pure observation to supervised patient care. GP mentors mentioned that independence grew over time, even if the full extent was not yet reached for all students due to the short duration and interruption by the COVID-19 pandemic. According to the mentors, activities were adapted to the student’s level of knowledge, previous professional experience, and concrete requests by the students. In the process, the GP mentors appreciated the student spending extra time on site, which lead to more collegial interactions and put the GP mentor similarly in the role of a learner. Some of the GP mentors tried to ensure that the students had their own patient whom they would see continuously, but most of them had difficulties in implementing this idea due to practical reasons.

*“We sit together in front of the patient, and I try to involve the students, so that they take part in the examination […] sometimes before the patient comes in, I talk briefly about the medical history with the students […] and when he is out, we talk together again either about the clinical picture or about the diagnosis. […] I think that all the students here are very independent, so they look at it, they are shown it once, [then] they also perform an ECG, they also do the lung function, and when they are in the laboratory, they take blood samples, we have also let them vaccinate as instructed.” (Physician No. 10, female)*

#### Communication and contact outside clinical visits

##### Communication and contact outside clinical visits from students’ perspectives

Examining how contact outside students’ clinical visits was structured, our results showed a broad range, from no contact to a regular exchange. Most students reported no or little contact between clinical visits, which was perceived as adequate by half of the students concerned. However, other students would have favored an intensification of contact. Only a few students described mutual contact outside the practice as being regular or happening on special occasions, e.g., on holidays or birthdays. Most communication happened via common smart phone messenger services and some even met in person at get-togethers concerning the practice or in a private setting. Especially for those who described contact as being very limited and being satisfied with this, get-togethers organized by the LeiKA-management team were welcomed.

*“So, mostly when I get in touch, of course [then we have contact]. So, he [my GP mentor] does not write me anything on his own initiative […]. But I’m trying to at least (—) well, depending on how stressful it is, at least once a month or so to get in touch. Just giving a brief update on how things are going and asking how things are going in the practice, whether they need any help or something” (Student No. 5, female)*

Uncertainty prevailed among students about whether they would stay in contact with their mentor after the official mentorship’s ending. Others were rather confident in the continuing nature of the mentorship in case of questions or concerns.

##### Communication and contact outside clinical visits from GP mentors’ perspectives

Many GP mentors reported no or little contact outside of clinical visits. Most perceived the lack of contact as an obstacle to a more intensive interaction, which would correspond with their ideas of mentoring. Other GP mentors had contact with their students on specific occasions, including inviting students to team parties, communicating on vacations and birthdays, or sending vacation greetings. A few of these mentors described an additional regular exchange and participation in the student’s life, e.g., by meeting for coffee or asking each other how the other was doing by means of messages. Common smart phone messenger services were the main medium used and contact was initiated by both the students and the GP mentors.

*“When I know it’s their [the students’] birthday, you send a few congratulations and so on. Or when they post vacation pictures in their status, then you write something about it, […] and then the students are invited in the summer and […] then we have a barbecue and sit together.” (Physician No. 8, female)*

#### Relationship level

##### Relationship level from students’ perspectives

When talking about the relationship to their GP mentor, a vast majority characterized their relationships as being merely professional, but perceived a difference compared to curricular internships regarding increased familiarity, working atmosphere, and GP mentors’ motivation. It was conceivable for several students to further develop the relationship on a personal level. A personal component with special interest in the other person was described by a smaller number of students. All students highly valued their GP mentor’s attention, whether shown in small gestures or by taking extra time for interactions and being available as a contact person. However, students rarely contacted their GP mentors outside of clinical visits and only a small group of students sought out personal advice from their GP mentor.

*“[the relationship is] kind of distant-friendly-professional. […] well, my GP mentor is a very nice, competent, but also a bit distant person, I think. So, it was a very professional teacher-student relationship from the beginning. And I also think it is not that much different now […]. Although I already have the feeling that, in the meantime, I simply know the whole practice better and that they also trust me in a certain way.” (Student No. 15, female)*

Regardless of the relationship type, several students mentioned difficulties at some point. Three students did not agree with their mentors’ behavior in certain situations, such as making racial comments or showing little empathy towards patients on a vegan diet, and felt the need to distance themselves from the situation. However, there was much uncertainty on how to address these concerns and one student reported a reluctance to contact the project administration.

##### Relationship level from GP mentors’ perspectives

When examining the relationship with their student, our results size the developmental nature of the interpersonal relationship. The GP mentors described the professional interaction as increasingly familiar and constructive, which also offered the opportunity for the GP mentor to step into the role of a learner. Several GP mentors expanded on this by characterizing the relationship as cordial and friendly. Many were nevertheless not satisfied and found the interaction difficult, saying that the connection was too loose and distant. Various reasons have been suggested, including that the project duration was still too short at the time of the interview, the GP mentors perceived the contact as too irregular and too little, and several criticized the lack of the student’s engagement in developing a close relationship. Even if the GP mentors stated being contacted by the student for advice, familiarity and trust differed between the mentee-mentor pairs, ranging from mere student-preceptor relationship to counseling in personal crisis. All GP mentors specifically asked could conceive of a continuous connection to their students even after the mentorship officially ended.

*“It is not like it is really a very close relationship, because you only have each other two, three times a year, or twice a semester. […] [The relationship is] very nice. Personally, as well, so we also meet for coffee sometimes […] He [the student] has also helped me with the vaccinations. […] So I think I am a friendly older colleague for the young man. […] Well with him, I know it is going to be that I will always follow him, where he is right now and if he calls and needs me, I will be there and if I ever need him, I will call him and then he will try to be there too.” (Physician No. 2, female)*

## Benefits

### Students’ benefits and perceived benefits for their GP mentor

All students described experiencing a variety of benefits from receiving mentoring. The familiarity with the GP mentor and the practice environment created a relaxed teaching atmosphere. Time for customization was shorter than in other teaching formats. In addition, students gained motivation and inspiration for their otherwise learning-intense studies and developed clear career goals. Further, students were faced with a great variety of responsibilities and additional tasks adopted by GPs, from social integration to psychosomatic care. Importantly, some students reported an increase in their appreciation for general practice and in their desire to pursue a career in this field. In addition, students appreciated insights into administrative and financial aspects of running a practice and received realistic impressions of everyday workload and financial gains. Almost all students saw the acquisition of skills as beneficial. They learned and improved their communication skills, took medical histories, and conducted physical examinations, but also honed medical assistant skills such as performing ECGs and drawing blood. Overall, the students valued the opportunity to transfer their knowledge gained from studying into practice. They found a contact person for medical and organizational questions and benefited from their connections by completing curricular teaching formats such as clinical clerkships in their GP mentor’s practice. Finally, some found a new family doctor for themselves or their relatives.

*“Well, I think, also more educational and I also get a lot more input […] with him [my GP mentor] in practice than I do in some other clinical clerkships, simply because I maybe have more confidence to ask something or to do something […] Well, it’s good for everyone to have someone who helps you a little bit through the difficult course of study and who tries a little bit to keep up the joy of the thing. By gaining a bit of practical experience and having contact with patients, and having more contact with patients than you might otherwise have during your studies.” (Student No. 5, female)*

When students were asked about their thoughts on the presumed benefits experienced by their GP mentors, many were unsure if mutual benefit was derived from the project. According to the students, GP mentors came into contact with academic medicine more easily. In addition, GP mentors were encouraged to critically question their own positions and to revise their own knowledge. Many students saw the interaction with the younger generation as beneficial to the GP mentors, mentioning that they often simply enjoyed being mentors. Students saw being able to improve their teaching skills as an additional benefit for the GP mentors. Further, students believed that the GP mentors appreciated being able to make an active contribution to the recruitment in general practice and that the students were able to support them in the practice.

### GP mentors’ benefits and perceived benefits for their students

Most GP mentors valued the professional exchange with the students, the current knowledge brought with the student, the critical self-reflection of their own knowledge and their everyday work, and the refreshing change that the student brought to their everyday work and team life. Many reported that they enjoyed being with young people, watching their development, and gaining insights into this generation’s lives. Further, GP mentors appreciated the opportunity to pass on their knowledge, values, and attitudes in a selected manner and thus were able to participate directly in the training of new physicians and to inspire them about general practice. Some found it helpful to have someone to ask when help was needed in practice.

*“That you always allow yourself to be questioned. That is a great benefit. That these questions also encourage you to structure your work better yourself or to defend why you are doing something […] and also that you, if I may say so, have guests coming to your home, you tidy up and so it is similar, […]you always have to make sure that you are up-to-date and that is extremely helpful. […] My personal gain, since I’ve been in the business for a long time now, is actually always that I look at it [the future] quite calmly, that I say: No, there are still very, very good students. All is not lost; the youth is not doing poorly, and the students are not stupid. The students are doing exactly the same thing that we were doing, not much has changed. That’s nice.” (Physician No. 3, female)*

When talking to GP mentors about their perceptions of students’ benefits, the mentors viewed the equalization of general practice with other curricular medical disciplines as a benefit for the students. The GP mentors believed that their students were able to gain insights into realistic general practice with all its advantages and disadvantages and that they acquired skills and were able to gain practical experience. The opportunity to experience aspects not taught at the university, such as the outpatient accounting system, procedures, and the complexity of treating patients in their psychosocial context were named as additional presumed benefits. The regularity and long-term nature of the mentorship gave the students the advantage of caring for patients over a long period of time, experiencing continuous learning progress, and transfer of medical values and attitudes, according to their GP mentors.

### Wishes and improvement suggestions

#### Students’ wishes and improvement suggestions

Students were asked about further wishes or suggestions of improvements. When asked explicitly, a major part expressed little about wishes for improvement, regardless of whether they stated any later during the interview. One student would favor an optimization of the matching process, and several would have appreciated a more structured introduction to the project and its objectives and teaching content for both students and GP mentors. Structured opportunities for exchange of expectations and feedback would be welcomed. Better communication of the expected teaching content to the GP mentors and improved integration of accompanying workshops and visit days were an important topic because some students felt that their GP mentors had little information on their learning level and on expected teaching content. Students wished that the first contact with the GP mentors would be supported and structured by the organizational team, because in some cases students had difficulties reaching the GP mentor by phone or the staff at the reception desk was not informed about project procedures. Many students would appreciate organized gatherings in terms of joint workshops or compulsory social events, to facilitate interaction with their GP mentor. Several students preferred less travel time to the practices to facilitate more spontaneous visits. There was disagreement among the students about the number of mandatory visit days. They knew that increasing the number would support the emerging relationship, but at the same time they were aware of the time burden, especially later in their studies.

*“I had recently thought about whether one could do interactive seminars, for example, like the ones we have in LeiKA anyway, maybe with the involvement of the GP mentors […]. If you would say, for example, I don’t know, for the LeiKA participants there are somehow two additional free days per semester, then I think it would be really, really great if you could increase the number of visit days. […] [And if the practice would be closer to Leipzig,] I’m pretty sure that I would just go there for an afternoon, […] and that just doesn’t work because if I always have to plan an hour for each way, then it’s only worth it if I get there early in the morning and then somehow drive back in the evening” (Student No. 7, female)*

When students were asked to describe what they believed GP mentors would wish for, the majority had difficulty doing so. Students named general matters of course – such as punctuality, friendliness – and limited GP mentor’s wishes to the teaching level.

#### GP mentors’ wishes and improvement suggestions

Several GP mentors wished to intensify the contact with the students and wished for more engagement and commitment by the students. More precisely, they asked for more timely planning and/or more regular contact initiated by the student. Additionally, shaping of and expectations towards mentoring relationships should be actively reflected and communicated by the students.

*“The student […] does contact me from time to time […] and I also contact her, […] but if you would put that in percentage terms maybe a bit in proportion, maybe 70:30, 80:20, that I always contact her more than she contacts me. Of course, it would be nice, […] but you can’t institutionalize that or things like that, […] but that there would be an initiative from the students as well, that […] would be nice.” (Physician No. 13, male)*

Regarding the project administration and structures, most GP mentors questioned if the two intended mandatory visit days per semester were sufficient to allow them to shape the mentorship according to their concept. Many wished to have more time to mentor students on additional days in their practice. Since GP mentors acknowledged the multiple obligations as a student, they proposed a better integration of the visiting days into the university schedule or official credits for them. Except for one GP mentor being limited in resources due to high patient load, most GP mentors stated having sufficient resources for additional practice visits. GP mentors also questioned whether the matching process could be optimized to allow mutual acquaintance and selection before allocation by the administration and whether the administration could monitor and encourage students’ regular participation in mandatory visit days more closely. Finally, some GP mentors considered it helpful to receive feedback and to have a more detailed teaching concept available for physicians and students.

*“The question of how intense the mentorship […] is structured, I think, only a few students envisioned it beforehand. I may be doing them an injustice, but I’m allowed to believe that. […] I am rather skeptical that this would really do any good, […] that the LeiKA management says: “You should contact your GP mentor. Have you gotten in touch?” We all have more than enough administration, it needs to work without a central office. […] [The two practice days are] not quite enough. It could be a bit more, but it has to fit into the overall concept, and despite all the valuation and importance we now assign to general medicine, it is not the only subject in medical school.” (Physician No. 12, male)*

When the GP mentors were asked about what they perceived their students wanted from them, most GP mentors hesitated. They believed that students wanted to be taught knowledge and in particular to be delegated tasks and be shown a wide range of medical activities. It was assumed that students would appreciate it if the GP mentor would be available and open – even to discuss personal issues such as work life balance – and to take time for students and their questions. Several GP mentors mentioned it is important to consider student’s daily routines and obligations so as not to expect too much.

## Discussion

### Summary of the main results

Our study aimed to examine the mentee-mentor relationships in a longitudinal extracurricular general practice teaching project by using qualitative semi-structured interviews. The benefits derived from the mentoring relationships were highly valued by participating students and GP mentors. The relationships differed in intensity and content according to individual needs. As the course progressed, many relationships evolved positively over time. More autonomy was transferred to the students and richer experiences became possible.

Expectations about the mentorship were often not clearly delineated by the mentors or the mentees, especially when it came to togetherness and interaction. Consequently, the configuration of the visit days was largely dependent on the physician’s initiative. The students often did not feel entitled to demand their GP mentor’s time and consideration and wished for independent medical tasks continuously adjusted to their current abilities. Conversely, the GP mentors criticized a lack of initiative from some of the students and wished that they would get more actively involved. Students, in contrast, wished for more guidance at the start of the project and joint events to deepen the relationship.

### Comparison with existing literature

#### Students’ and GP mentors’ mentoring relationship, definition, and roles

The nature and depth of the student-physician relationship was diverse. It became apparent in both groups that LeiKA is not a classic mentoring program but contains components of teaching, precepting, and mentoring. Students’ main motivations for participating in the project (i.e., early practical experiences, contact with patients and GPs, and early insights into ambulatory care [27]) could have been covered by mere preceptorship. On the other hand, several participants described characteristics of their relationship that would be most consistent with ‘classic’ mentoring. In addition, relationships evolved and changed their characteristics, depth, and proximity over time.

Several authors distinguish between preceptorship and mentoring, while the second being described as focusing on personal, scholarly, and career development; long-term nature; and taking place outside the work environment [41]. By contrast, Radha Krishna et al. recently reconceptualized mentoring and suggested that all four aspects (i.e., role modeling, teaching/tutoring, coaching, and supervision) can be part of mentoring. However, the proportional composition of these aspects depends on the people involved and, based on this, the complex mentee-mentor relationship can develop through an individualized, holistic, and long-term approach [42]. This definition corresponds well to the relationships we explored and our findings, showing that the individual shaping of the relationship was widely varied, corresponding to different personalities, demands, and needs.

The multiple roles senior doctors adopted during the project have also been highlighted by Rodríguez et al., indicating that being a mentor is only one role besides emotional support, role model, and teacher [34]. As in our study, it became clear, however, that many GP mentors and students seemed to enter the project without having a clear idea of the roles they would play and the tasks they would take on. For this purpose, mechanisms and formats could be found to encourage timely and regular reflection and exchange on the topic of mentorships. This could be supported by information on what mentorship has to offer and how to technically make the most of it for the satisfaction of both parties.

#### Communication of expectations

In our study, we found a wide variance in the communication of expectations between students and GP mentors, ranging from no prior expectation sharing to detailed discussions about mutual goals. The exchange of mutual expectations is an essential step to enable richer experiences for students [18] and was identified as an important starting point for the optimization of corresponding programs in several studies [26,34,43,44]. While fixed occasions and guidance for initial acquaintance and exchange about the structuring of the mentoring sessions are an integrative component of some projects [43], others report that mentors used their own checklist for their initial meetings [36].

Regardless of the extent of initial training and structuring exchange and feedback, many authors reported the need for optimization regarding pre-education and training of both parties [21,23,26,36,43]. Specifically, information on the aim and content of mentoring in general and the respective programs in particular for all participating parties have been proposed as suitable, though maybe not sufficient, tools to achieve a better alignment of expectations and to avoid disappointments. Such information should include the following: reflection on personal goals and roles [26,43], provision of communication and feedback skills [36], peer coaching and supervision of mentoring sessions [21], and written guidelines and ‘codes of conduct’ [21,23,26,43]. Because our project does not include structured feedback talks so far, this study shows in agreement with existing literature that this is a point to be prioritized in further planning and deserves the deployment of considerable financial and human resources as proposed by Rodriguez et al. [34]. For many years now, most new teaching physicians have received didactic training as part of their qualification course, including the topic of feedback. Maintenance and expansion of these courses on a regular and possibly mandatory basis seems thus advisable. In addition, integration of regular and more formalized feedback opportunities for GP mentors and students, both in the project course and within mandatory visit days, might be suitable tools.

### Practical realization of teaching

Students appreciated that they were able to acquire a variety of skills and gained realistic insights into everyday practice life with its positive and negative aspects. They took on a variety of roles and most achieved the desired level of independence in treating patients under supervision.

Our results highlighted a strong dependence of successful teaching moments on the motivation, engagement, and creativity of the teaching physicians, while students perceived substantial barriers in demanding concrete content and shaping the teaching situation. Our results are in line with findings from Fernald et al. in a similar program, showing that students valued being ‘pushed’, both by being allowed a high degree of autonomy in seeing patients and by being asked questions [18], which stimulates their learning process.

The important role of the GP in ambulatory care teaching situations has been conceptualized in a review by Park et al., assigning the role of a broker to the GP, who provides the conditions for successful learning by contextualizing the situation with the student. The authors described the GP’s task as being in charge of ‘scaffolding for the learning by understanding and ensuring relevance to the medical curriculum, and focusing and contextualising the student’s existing knowledge […] and providing a structured timetable’ [45]. Other factors for preceptors’ effective teaching and learning included motivation and enthusiasm [15,16], allowing students autonomy and responsibility in patient encounters [18,19], and challenging students to leave their comfort zone [18] in an encouraging and supporting way [15,18].

We conclude from our students’ comments that most preceptors enabled stimulating learning situations, however, with limitations regarding knowledge about the medical curriculum and partly the students’ learning level, both of which have also been described as barriers for learning in the literature [15,19]. This again highlights the need for more intensive information and training for preceptors, especially regarding learning objectives, and a closer link with the program administration.

This is, of course, limited by personnel, time, and financial resources.

Further, studies investigating the relationships between student and preceptor found that a respectful and trusting relationship is fundamental for students to be given the desired autonomy in patient encounters [18,20]. We see indications in our results that the design of the project over several years could particularly contribute to the development of a trusting relationship, though the small number of required practice days turned out to be a barrier. This is supported by students’ statements, indicating that those who completed long-term stays in their GP mentors’ practices achieved an intensification of their relationships and reported experiencing greater autonomy.

### GP mentors’ and students’ benefits

The GP mentors reported diverse benefits from participating in the project, such as constant knowledge updates and self-reflection of current knowledge and habitual procedures. Our findings are in line with the review by Park et al., reporting cognitive, behavioral, and emotional benefits for GP teachers through their involvement in undergraduate medical education [45]. Among those, and beyond, are the following: contact with academic medicine/gaining new knowledge [9,24,45], improved teaching skills [9,24,45], relationship/work with the next generation of physicians [9,24,46], conveying complexity of and experiences in general practice [47], joy/personal fulfillment [23,26,45–47], and variety in their work [45].

From the comments of the GP mentors in our interviews, it became clear that our GP mentors also desired to convey a positive image of general practice and to contribute to the recruitment of the next generation of physicians. Compared with the literature, this topic was identified as newly emerging in our study, which may be due to the pressing issue of GP shortages. Communicating the variety of benefits GP mentors receive might be helpful for recruiting and sustaining future teaching physicians.

Students were well aware of their own benefits. In contrast, they were often unaware of the reciprocal benefits their GP mentor received from the project. This might have contributed to the students’ reluctance to proactively express their expectations to their GP mentor. Conveying the extent of the benefits for GP mentors to students might be helpful for encouraging students to adopt a more active role.

Students especially benefited from the familiar environment in the practice and from the opportunity for hands-on tasks and experiences. Both the close contact to patients and insights in the practice management were perceived to be valuable and might have contributed to the increased attractiveness of the profession for some students. The wide variety of benefits to students is congruent with the existing literature [18,26,35,45] and therefore will not be further discussed. The LeiKA project was based on the well-known benefits that longitudinal experiences bring to students. Our results show that this concept has successfully achieved its goal.

### Additional wishes for improvement

When both parties were asked about ideas to support the mentorships, multiple suggestions were expressed. Many GP mentors identified a lack of shared time as a barrier and would welcome an increase in the number of regular visits albeit being aware of the students’ everyday obligations and their involvement with the curricular studies. Students were equally ambiguous of the increase in mandatory visit days. Other longitudinal general practice programs worldwide reported a broad range in meeting frequency, ranging from few occasions per year to one per week [18,34,48–50]. In some programs, students spend regular teaching time with their senior physician; other programs like ours demand time spent ‘on top’ of the curricular content (e.g., three yearly training evenings for GPs [49]; workshops, electives, and didactic dinner meetings for students [48]).

However, time investment remains a controversial topic in many programs [31]. Time for effective teaching and mentoring on the part of the preceptors has been identified as a critical factor in teaching in general and in longitudinal programs specifically [9,16,46,51]. Rodríguez et al. reduced mandatory teaching periods from 20 to 16 days per year and increased their flexibility after evaluation in a one-year longitudinal preceptorship [34]. In our project, we decided not to change the number of 4 days per year. Instead, additional meetings tailored to the participant’s individual needs were supported and students were encouraged to additionally complete their (curricular) general practice clerkship in their GP mentor’s practice whenever possible.

Further, several participants, mainly the preceptors, wished for an optimization of the matching process. There is evidence that a congruence of values, personal interests, and goals can contribute to a better relationship [23,26,42], but not all these aspects have been considered in our program due to a limited number of GPs. However, we observed over time that gender congruence seems to play a role and female students are more likely to request a female GP mentor. Our impressions are supported by the literature. Gender congruence of mentor and mentee could strengthen the mentoring relationship [26] and female mentors could have a special significance for female students to show the compatibility of work and family commitments [22]. This topic opens up room for improvement, discussion, and further research.

Additional ideas for improvement expressed by the participants such as joint events, regular feedback opportunities, and a closer link between practical days and project content have been discussed elsewhere in this section.

### Strengths and limitations

This study fills an important knowledge gap by providing detailed insights into student-mentor relationships in longitudinal general practice recruitment programs, including both student and physician perspectives, development over time, and areas for improvement. However, some limitations should be considered when interpreting the results. First, participation was voluntary. Students willing to participate might have been those with a higher commitment to our project and/or general practice and we might unintentionally miss students who were less enthusiastic, involved, or satisfied. With this in mind, we tried to maximize the heterogeneity of our sample in advance by considering potentially relevant GP and student characteristics when selecting participants. Furthermore, students who dropped out of our project prematurely were not considered. Their views and characteristics are currently under exploration in a separate study.

Second, we interviewed students and preceptors cross-sectionally, which impedes a direct observation of the development and change of the mentoring relationships. However, to get an idea of the developing nature, the participants were additionally asked to describe their perceptions in retrospect.

Third, due to the COVID-19 pandemic, there was an interruption of the learning progress and of the development of the mentoring relationships, as practice visits were stopped in the lockdown period between March 2020 and October 2021.

Further, the previously described specificity of our project compared with other longitudinal general practice programs and the fact that the study was conducted only at one German medical school may limit the generalizability of the findings.

### Conclusion and implications for practice and further research

With this study, we gained detailed insights into and understanding of the nature of developed relationships between students and GP mentors in an extracurricular multi-year general practice teaching project. The design of the mentoring varied individually. A high variance was apparent in the shaping of the relationship level, which was adapted to individual needs. The design of the mandatory visit days was largely dependent on the initiative of the physician. As the course progressed, the relationship evolved over time, and richer experiences became possible through the trust and familiarity that developed. Although the experience was well adopted and highly valued by both parties, potential for improvement was revealed.

The following needs for program improvement emerged from the interviews and could contribute to the planning and further development of similar projects: 1) concrete familiarization of both GPs and students with the goals of mentoring, time commitment, and rules of conduct; 2) sufficient support and training for participating GPs to be able to implement their function, e.g., communication of teaching content, intensified didactics training, firmly implemented framework for regular and mutual feedback between students and GP mentors; 3) formalization and support on the part of the administration to facilitate the initial contact between mentor and mentee; 4) encouragement and support of additional (teaching) time spent together, individualized timing and intensification if desired; and 5) emphasis on the matching process with the best possible inclusion of individual wishes and characteristics, considering the available resources of teaching physicians.

Long-term studies should evaluate the impact of these mentorship programs on the career choices of medical students. Future research with longitudinal data regarding project graduates’ career choices is needed to address this issue.

## Data Availability

Data are available from the authors upon reasonable requests.

## Appendix 1: Translated interview guideline

**Translated interview guideline – Students**

1. Introduction
  - introduction of the interviewer
  - presentation of the study’s purpose
  - interview procedure
  - information on data protection, recording and anonymized publication
2. Warm-up question
  - What attracted you to a long-term mentorship with a GP?
3. Main questions
  - What expectation did you initially have of the mentorship to your GP mentor?
  - To what extent were these expectations initially communicated with your GP mentor?
  - How would you currently describe the relationship with your GP mentor?
  - To what extent do you have contact in addition to your regular visits?
  - To what extent have your initial expectations of the mentorship been met?
  - How would you define mentorship as part of the *project X*?
  - To what extent does this correspond to the concept of a mentorship for you?
  - Where do you see peculiarities and differences between your GP mentor and other physicians from different internships?
  - To what extent do you currently benefit from this mentorship?
  - What do you wish for from your GP mentor as part of the *project X*?
  - What do you think, your GP mentor would like you to do?
  - From your point of view, how could the relationship between you and your GP mentor be supported?
  - Where do you see the overall profit from a long-term mentorship between student and GP for the students?
  - Where do you see the overall profit from a long-term mentorship between a student and a GP for GP mentors?
4. Wrap-up question
  - Would you like to add something? Is there anything important to you that we haven’t talked about yet?
5. Termination
  - Thank you for your participation. Have a nice day.

**Translated interview guideline – GP mentors**

1. Introduction
  - introduction of the interviewer
  - presentation of the study’s purpose
  - interview procedure
  - information on data protection, recording and anonymized publication
2. Warm-up question
  - What attracted you to a long-term mentorship with an interested student?
3. Main questions
  - What expectation did you initially have of the mentorship to your student?
  - To what extent were these expectations initially communicated with your student?
  - How would you currently describe the relationship with your student?
  - Where do you see your tasks in the role of a GP mentor?
  - To what extent do you have contact in addition to your student’s regular visits?
  - To what extent have your initial expectations of the mentorship been met?
  - How would you define mentorship as part of the *project X*?
  - To what extent does this correspond to the concept of a mentorship for you?
  - Where do you see peculiarities and differences between your student and other students from different internships?
  - What do you wish for from your student as part of the *project X*?
  - What do you think, your student would like you to do?
  - From your point of view, how could the relationship between you and your student be supported?
  - Where do you see the overall profit from a long-term mentorship between student and GP for the GPs?
  - What is your personal benefit from this mentorship?
  - Where do you see the overall profit from a long-term mentorship between a student and a GP for students?
4. Wrap-up question
  - Would you like to add something? Is there anything important to you that we haven’t talked about yet?
5. Termination
  - Thank you for your participation. Have a nice day.
